# Metagenomic and Targeted Next-Generation Sequencing in Infectious Disease Diagnostics: Current Applications, Challenges, and Future Perspectives

**DOI:** 10.3390/diagnostics16070991

**Published:** 2026-03-25

**Authors:** Rong Rong, Yuni Long, Yujing Li, Lanxi Lin, Jie Yang, Ziqi Hu, Dayue Liu, Peisong Chen

**Affiliations:** 1 Department of Nosocomial Infection, The First Affiliated Hospital, Sun Yat-sen University, Guangzhou 510080, China; rongr@mail.sysu.edu.cn (R.R.); liyujing3@mail.sysu.edu.cn (Y.L.); linlanxi@mail.sysu.edu.cn (L.L.); 2 Zunyi Medical University, Zhuhai Campus, Zhuhai 519000, China; 3 Medical Affairs Department, The First Affiliated Hospital, Sun Yat-sen University, No.58 Zhongshan Er Road, Guangzhou 510080, China; yjie2@mail.sysu.edu.cn (J.Y.);; 4 Department of Laboratory Medicine, The First Affiliated Hospital, Sun Yat-sen University, No.58 Zhongshan Er Road, Guangzhou 510080, China; 5 Department of Laboratory Medicine, People’s Hospital of Boluo County, Huizhou 516100, China

**Keywords:** metagenomic next-generation sequencing (mNGS), targeted next-generation sequencing (tNGS), infectious disease diagnostics, antimicrobial resistance (AMR), clinical metagenomics

## Abstract

Metagenomic and targeted next-generation sequencing (NGS) technologies are rapidly transforming diagnosis and management for infectious diseases. This review comprehensively examines the current applications of metagenomic NGS (mNGS) and targeted NGS (tNGS) in clinical microbiology, highlighting their roles in pathogen detection, antimicrobial resistance profiling, virulence characterization, and outbreak investigation—particularly in complex cases such as pneumonia, critical illness with pulmonary infections, and pediatric acute respiratory illnesses. We discuss the diagnostic performance, advantages, and limitations of these approaches, including challenges related to sensitivity, specificity, standardization, bioinformatic complexity, and cost-effectiveness. Furthermore, we explore emerging opportunities for integrating NGS-based surveillance with public health strategies, such as wastewater epidemiology, to monitor healthcare-associated infections (HAIs) and antimicrobial resistance (AMR) at the population level. Finally, we outline key steps needed to translate these powerful genomic tools from research settings into routine clinical and public health practice.

## 1. Introduction

Infectious diseases remain one of the leading causes of human morbidity and mortality worldwide, posing a persistent threat to global public health. Rapid and accurate identification of pathogens from clinical samples is crucial for guiding appropriate treatment strategies and improving patient outcomes. Traditional diagnostic methods, including microbial culture, antigen–antibody testing, and polymerase chain reaction (PCR)-based assays, have served as the cornerstone of clinical microbiology for decades. However, these conventional approaches face significant limitations that often impede timely and effective diagnosis [1,2].

Microbiological culture, despite being considered the gold standard, requires at least 24 h for pathogen growth, followed by an additional 1–2 days for antimicrobial susceptibility testing [3,4]. This extended turnaround time frequently necessitates the initiation of empirical broad-spectrum antimicrobial therapy, which can lead to suboptimal treatment outcomes, unnecessary antibiotic exposure, and increased risk of antimicrobial resistance (AMR). Furthermore, culture-based methods are inherently limited in their ability to detect fastidious, slow-growing, non-culturable, or anaerobic pathogens, resulting in a diagnostic gap in approximately 50% of infected patients. Many clinically relevant microorganisms, including certain respiratory pathogens, remain difficult or impossible to culture in vitro, significantly compromising diagnostic accuracy.

In patients with severe infections, including pneumonia, bacteremia, central nervous system (CNS) infections, and other deep-seated infections, early detection of the causative microorganism is essential for instituting timely clinical interventions and administering appropriate antimicrobials. However, the use of broad-spectrum antibiotics prior to pathogen identification, coupled with the invasive nature of obtaining samples from deep infection sites, often confounds specific diagnosis. Additionally, traditional diagnostic methods exhibit low throughput and limited capacity to simultaneously detect multiple pathogen types, including bacteria, viruses, fungi, and parasites, within a single test.

Emerging rapid diagnostic technologies, such as adenosine triphosphate (ATP) bioluminescence and multiplex PCR assays, offer faster results but still face substantial limitations. ATP bioluminescence, while rapid and cost-effective, lacks specificity as it detects both microbial and non-microbial ATP, making it more suitable for surface cleanliness monitoring than for pathogen detection [5,6]. Multiplex PCR-based assays, though capable of detecting multiple predetermined targets, require prior knowledge of suspected pathogens and cannot identify unexpected or novel microorganisms.

The major mainstream high-throughput sequencing technologies include metagenomic next-generation sequencing (mNGS) and targeted next-generation sequencing (tNGS). mNGS is typically detected using second-generation sequencing (SGS) or third-generation sequencing (TGS) platforms, representing a transformative diagnostic paradigm that addresses many of these limitations. For example, microbiological culture methods typically require 48–72 h to obtain the results [7], while the turnaround time of conventional mNGS is 24 h. By directly analyzing all microbial and host genetic material in clinical specimens without the need for prior cultivation or pathogen-specific amplification, mNGS provides a culture-independent, hypothesis-free, and unbiased approach to pathogen identification [8]. This revolutionary technology enables simultaneous detection of virtually all known pathogens—including bacteria, viruses, fungi, parasites, and even non-culturable organisms—in a single comprehensive assay. Beyond pathogen identification, mNGS can characterize antimicrobial resistance genes, virulence factors, and provide genomic epidemiological insights, making it particularly valuable for diagnosing critical, complex, rare, and emerging infections where conventional methods fail [8,9].

tNGS is an advanced diagnostic technique that utilizes high-throughput sequencing platforms combined with specific primer sets or probe panels to enrich and sequence nucleic acids from a predefined list of pathogens. In clinical testing, tNGS is primarily used to identify pathogens and antibiotic resistance genes in patient samples [10]. Unlike the unbiased approach of mNGS, tNGS focuses on specific microorganisms of interest [11], which enhances sensitivity and specificity, particularly for detecting low-abundance pathogens in clinical samples. Its targeted approach offers a practical and clinically valuable alternative to mNGS for the rapid diagnosis of infections.

Despite its remarkable potential, several technical and practical challenges remain in the clinical implementation of mNGS. For handling of commensal organisms, false positives and false negatives still remain significant issues. The predominance of host DNA in clinical samples often obscures pathogen detection, necessitating host nucleic acid depletion strategies [12]. Environmental and reagent contamination can lead to false-positive results, while the complexity of bioinformatics analysis requires sophisticated computational tools and specialized expertise for accurate data interpretation [9]. In laboratory phase, loss of microbial nucleic acids may result in false negatives. Meanwhile, mNGS shows lower sensitivity for RNA viruses’ detection. Additionally, considerations regarding cost-effectiveness, turnaround time, standardization of protocols, and establishment of clinical interpretation guidelines must be addressed to facilitate widespread adoption in routine diagnostic practice.

This review aims to comprehensively evaluate the current state of metagenomic sequencing in infectious disease diagnostics. We will examine its fundamental principles, technical platforms, and clinical applications across various organ systems, highlighting its comparative advantages over traditional methods. Furthermore, we will explore the expanding role of metagenomics beyond individual patient diagnosis into the realm of public health, specifically its innovative application in the surveillance of healthcare-associated infections (HAIs) and AMR through wastewater-based epidemiology. Finally, we will discuss existing challenges and future directions for translating this powerful technology into routine clinical care and public health practice.

## 2. Next-Generation Sequencing Technologies

mNGS and targeted next-generation sequencing (tNGS) are the two major mainstream high-throughput sequencing technologies in the field of molecular diagnosis of infectious diseases at present.

### 2.1. mNGS: Overview, Technological Advancements, and Challenges

mNGS is a cutting-edge sequencing method that enables the unbiased, comprehensive detection of a broad range of microorganisms within a sample. This technology does not rely on predefined pathogen targets, providing the flexibility to identify bacteria, viruses, fungi, and parasites, including rare or atypical pathogens that might be missed by traditional diagnostic methods. mNGS is particularly beneficial for diagnosing complex or mixed infections, where conventional techniques fall short [13].

The mNGS process works by fragmenting the DNA of both microbial and human sources in a sample. These fragments are then used to generate libraries, which are sequenced to produce individual reads. The resulting data is then compared against reference databases, such as GenBank and RefSeq, to determine the microbial species and their abundance within the sample. This method offers a far more comprehensive genomic analysis than culture-based techniques, which are limited to culturable organisms [2]. Moreover, mNGS not only identifies microbial species and strains but also provides an unbiased snapshot of the microbial community at the time of sequencing, capturing the genetic repertoire present in the sample—including metabolic potential, virulence factors, and antimicrobial resistance genes—without the need for prior culture or pathogen-specific amplification [14].

#### 2.1.1. Sample Types and Nucleic Acid Selection Strategy

mNGS can be used for bacterial, fungal, parasitic, and various viral infections and is primarily a sequence alignment process for nucleic acids extracted from infected samples. Due to fundamental differences in the biochemical properties of DNA and RNA, distinct extraction and library preparation methods are required for each nucleic acid type. Specifically, RNA must be reverse-transcribed into complementary DNA (cDNA) prior to sequencing. Therefore, the choice of testing strategy should be guided by the suspected pathogen type: DNA sequencing is recommended when infections caused by DNA-based pathogens—such as bacteria, fungi, DNA viruses, or parasites—are suspected. Though there are inevitable limitations of RNA sequencing, such as its higher cost, complexity and the requirement for sample freshness, RNA sequencing is recommended if RNA viral infection is suspected. Co-testing of DNA and RNA is recommended if it is not possible to determine which type of viral infection is involved [15].

In addition, the diagnosis of infectious diseases requires that specific samples must first be collected from the site of primary infection before being processed preprocessed. For example, bronchoalveolar lavage fluid (BALF) and sputum are typically recommended for lung infections, while cerebrospinal fluid (CSF) is recommended for CNS infections. While library construction, sequencing, and bioinformatics analysis can be the same for different samples, pretreatment and nucleic acid extraction vary depending on the sample source [15].

#### 2.1.2. Technological Platforms: SGS vs. TGS

mNGS employs both second-generation sequencing (SGS) and third-generation sequencing (TGS) technologies, which differ primarily in their sequencing read lengths. SGS technologies, such as Illumina and Ion Torrent, are known for their high throughput, precision, and affordability, though their shorter read lengths can necessitate more complex data assembly [16]. The Illumina error rate is 0.1% [17], while the Ion Torrent error rate is even lower than that [18]. These platforms require PCR amplification during library preparation, which can introduce biases such as GC bias, amplification errors, and loss of native modification information. In contrast, TGS platforms, including Oxford Nanopore and PacBio, generate longer reads (often exceeding 10 kb), which simplify genome assembly and provide a more accurate representation of microbial communities. For PacBio, traditional long reads had high error rates (~14%), but the HiFi sequencing mode on the Sequel II system achieves accuracy of up to 99.8% [17]. The accuracy of Oxford Nanopore can reach 99.999% [17]. A key distinction of TGS is its ability to sequence DNA or RNA molecules directly without prior amplification, preserving native base modifications and avoiding the biases introduced by PCR enrichment This ability to sequence DNA or RNA directly without amplification marks a significant advancement in metagenomic sequencing, facilitating more accurate microbial community analysis [19,20].

### 2.2. tNGS: Advancements in Pathogen Detection

tNGS represents a strategic evolution in pathogen detection methodology, wherein specific microbial nucleic acid sequences are selectively enriched prior to high-throughput sequencing. Unlike the untargeted approach of mNGS, tNGS employs predetermined primer or probe systems to specifically amplify or capture pathogen nucleic acids of clinical interest, thereby substantially increasing the proportion of target sequences within the target panel [21]. The fundamental principle underlying this technology involves concentrating sequencing resources on clinically relevant pathogens while minimizing interference from host background nucleic acids and non-target microorganisms.

The application of NGS technology to infectious disease diagnostics traces back to the early 2010s, when mNGS was initially employed for viral pathogen detection [22]. However, clinical implementation of mNGS has revealed several challenges, including high levels of human genomic DNA contamination, difficulties distinguishing colonizing organisms from true pathogens, and substantial testing costs [23]. In response to these limitations, targeted sequencing strategies emerged as cost-efficient alternatives. While the core methodologies of tNGS—utilizing either PCR amplification or hybridization-based capture for target enrichment—have remained relatively consistent since the 2010s [24,25], the scope of target coverage has expanded dramatically from single-locus assays (such as 16S rRNA, 18S rRNA, or ITS region sequencing) to highly multiplexed panels capable of detecting hundreds or even thousands of pathogen targets simultaneously [21]. This approach significantly improves the sensitivity and specificity of pathogen detection, particularly for pathogens that are difficult to culture or for mixed infections.

Due to lower costs, tNGS is suitable for known or limited pathogen spectra. Conversely, in emerging and rare infections, especially when conventional diagnostic methods yield negative results, tNGS has disadvantages compared with mNGS.

#### 2.2.1. Technological Platforms: mp-tNGS and hc-tNGS

tNGS technology comprises two principal technical platforms: multiplex PCR-based targeted NGS (mp-tNGS) and hybrid-capture-based targeted NGS (hc-tNGS), each employing distinct enrichment strategies with specific advantages and suitable applications (Table 1).

##### Multiplex PCR-Based Targeted NGS

mp-tNGS utilizes multiple primer sets in a single reaction to simultaneously amplify numerous pathogen-specific targets. Panel design integrates information from published literature and epidemiological data to identify clinically relevant pathogens [26,27,28]. A typical respiratory pathogen panel encompasses approximately 200 target species commonly encountered in clinical practice [29]. Reference databases are curated from authoritative sources such as NCBI RefSeq [30] and GenBank [31], with systematic quality control measures including removal of redundant sequences, filtering of contaminating sequences, and phylogenetic analysis for accurate taxonomic classification [32]. For antibiotic resistance genes (ARGs) and virulence factors identification, the reads were usually aligned to the reference based on Comprehensive Antibiotic Research Database (CARD) and Virulence Factors Database (VFDB) [33].

Primer design follows established molecular biology principles, with parameters optimized for melting temperature consistency (approximately 60 °C), balanced GC content (40–60%), and appropriate primer length (18–26 base pairs) to ensure efficient multiplex amplification. For high-priority pathogens requiring subtyping capability, multiple primer sets are incorporated to ensure robust detection and characterization. Following nucleic acid extraction, samples undergo multiplex PCR amplification, library preparation, and sequencing on high-throughput platforms, with bioinformatics pipelines comparing sequencing data against reference databases to identify and quantify detected pathogens [21].

##### Hybrid-Capture-Based Targeted NGS

hc-tNGS builds upon standard mNGS library construction workflows by incorporating a probe hybridization step to selectively enrich microbial sequences. Comprehensive probe panels are designed to target species-specific genes, conserved microbial sequences, antimicrobial resistance determinants, and virulence factors across thousands of clinically relevant pathogens. Following library preparation, probe hybridization selectively captures target sequences, with captured fragments subsequently sequenced and analyzed using similar bioinformatics approaches to mp-tNGS. It can simultaneously enrich thousands or even tens of thousands of targets, making it suitable for large panels and complex samples [34,35].

Optimization studies have established optimal reaction parameters, including probe concentration, hybridization temperature, and reaction duration, to balance detection performance with clinical requirements for rapid turnaround times. While extended hybridization periods may yield optimal sensitivity, abbreviated protocols (30 min hybridization) provide acceptable performance while meeting clinical workflow demands [21,36]. hc-tNGS offers broader target coverage compared to mp-tNGS, making it particularly suitable for scenarios where the range of potential pathogens is uncertain or when comprehensive microbial genomic information is required [21,36].

The choice between mp-tNGS and hc-tNGS platforms depends on specific diagnostic requirements and resource considerations. mp-tNGS excels in rapid, precise detection of well-defined pathogen panels, making it ideal for scenarios with clear diagnostic targets and requirements for expedited reporting. hc-tNGS demonstrates advantages in breadth of target coverage, simultaneously enriching diverse pathogen sequences, thereby proving suitable for complex infections or situations requiring comprehensive pathogen profiling.

## 3. Clinical Applications of mNGS and tNGS in Infectious Disease

mNGS and tNGS represent two major high-throughput technologies in the molecular diagnosis of infectious diseases in recent years. Their clinical applications span multiple organ systems and infection types, providing powerful tools for pathogen detection, antimicrobial resistance profiling, and precision medicine.

### 3.1. Central Nervous System Infections (CNSI)

#### 3.1.1. Application of mNGS in Central Nervous System Infections

##### mNGS in Common CNS Infections

In a multicenter prospective study of 204 hospitalized patients with meningitis or encephalitis, mNGS identified additional pathogens in 22% of confirmed infections missed by standard tests, and the results directly changed therapy in 54% of these cases [37]. Another multicenter cohort including 213 patients with infectious and non-infectious CNS disorders reported a 57% positive detection rate in confirmed infections. The diagnostic performance varied by etiology, with areas under the curve (AUCs) of 0.659 for viral, 0.619 for tuberculous, and 0.846 for bacterial meningitis; sensitivity reached 73.3% for bacterial, 76.9% for cryptococcal, and 80% for *Aspergillus meningitis* [38].

Clinical translation of CSF mNGS is now underway worldwide. The University of California, San Francisco (UCSF) Clinical Laboratory assay demonstrated 73–92% sensitivity and 96–99% specificity [39]. In the multicenter Precision Diagnosis of Acute Infectious Diseases (PDAID) study, mNGS achieved 80% positive and 98% negative percent agreement with conventional CSF detection methods, including culture, antigen testing, PCR, or confirmatory mNGS, increasing overall infectious diagnoses by 22%. Of the 13 infections identified exclusively by mNGS, 8 directly altered clinical management. However, mNGS failed to detect approximately 45% of all infections, largely because some pathogens were detectable only by serology such as *West Nile virus* or *Treponema pallidum*, some were compartmentalized lesions such as abscesses, and others produced DNA titers below the sequencing threshold. Occasional false positives from Pantoea, *Staphylococcus aureus*, or *Streptococcus agalactiae* reflected environmental or commensal contamination, emphasizing that clinical reasoning and confirmatory testing remain essential [39].

Building on these data, a 2024 evidence review further concluded that mNGS currently represents the most promising diagnostic platform for CNS infections, outperforming molecular panels such as the BioFire FilmArray [40]. In a multicenter study of 58 confirmed meningoencephalitis cases—including stem cell and solid organ transplant recipients—19 (33%) infections were detected by both conventional methods and mNGS, 26 (45%) by conventional testing alone, and 13 (22%) by mNGS alone, achieving a Standards for Reporting of Diagnostic Accuracy Studies (STARD) score of 29/30 [37]. These results provide strong evidence of the stand-alone diagnostic capability of mNGS in selected or elusive CNS infections, while current evidence still supports its combined use with traditional assays. The review also emphasized the need for larger studies on CNS fungal infections to refine the clinical utility of mNGS [40].

In pediatric bacterial meningitis, mNGS reached 73.1% sensitivity and 88.1% specificity for *Streptococcus pneumoniae* and served as the sole positive method in some cases [41]. The technology also remains highly effective in situations where patients have already been treated with antibiotics, which can inhibit pathogen growth in cultures and lead to false negative results despite ongoing infection [42]. Earlier CSF sampling (<14 days after onset) yielded significantly higher read counts, confirming that early sampling improves diagnostic yield. In a single-center cohort of 248 suspected CNS infections, mNGS achieved 90% sensitivity in untreated culture-positive patients and 66.7% in those who had received empirical antibiotics. In culture-negative cases, mNGS detected 48 additional bacteria and fungi, outperforming culture both in treated (34.45% vs. 7.56%) and untreated (50.00% vs. 25.00%) groups [43]. High CSF white cell counts (>300 × 10^6^/L), protein > 500 mg/L, and low glucose ratios ≤ 0.3 were linked to higher mNGS positivity. In such culture-negative cases, mNGS can still detect microbial nucleic acids from non-viable or growth-inhibited organisms, providing crucial etiological information that would otherwise be missed. During the inflammation progression, the proportion of pathogen-specific sequencing reads may increase with inflammation, suggesting a role in dynamic disease monitoring. For tuberculous meningitis (TBM), mNGS also demonstrated substantial advantages. Detection of the *Mycobacterium* tuberculosis complex reached 78.3%, with 66.7% sensitivity—higher than smear (33.3%), PCR (25%), or culture (8.3%)—and 100% specificity. Combined mNGS and conventional testing raised total yield to 95.7% [44]. This capability enables clinicians to de-escalate empirical broad-spectrum therapy, transition to targeted antimicrobial treatment, and potentially improve patient outcomes while reducing unnecessary antibiotic exposure.

##### mNGS in Viral CNS Infections and Limitations

mNGS has proven effective in detecting viral CNS infections, including *herpesviruses* and other neurotropic viruses. Early work identified herpes simplex virus type 1 (HSV-1), HSV-2, and *varicella-zoster virus* (VZV) from the CSF of four patients with viral meningoencephalitis, with read counts ranging from 144–44,205 and coverage between 12 and 98%; PCR confirmed the findings [44]. In immunocompromised hosts, mNGS identified JC virus as the causative agent of progressive multifocal leukoencephalopathy (PML) in an HIV-positive patient [45], and detected 97,248 VZV-specific reads (99.91% coverage) in another HIV-infected patient lacking the typical rash, confirming fulminant VZV encephalitis [46]. Additionally, a case of human pseudorabies virus infection following exposure to contaminated hog-farm sewage was diagnosed using high-throughput sequencing and confirmed by PCR [47].

Although mNGS offers broad viral detection, its diagnostic yield for viral encephalitis remains lower than for bacterial or fungal infections. Therefore, it is advised that RT-PCR is adopted in these situations due to its sensitivity and cost effectiveness in comparison with mNGS. The limitations are primarily due to the degradability of RNA sequencing, low viral titers, and delays in sample collection. Studies show that mNGS positivity declines with prolonged treatment duration, emphasizing the importance of early sampling and combined DNA/RNA sequencing strategies to enhance viral detection [38].

##### mNGS in Rare and Culture-Negative CNS Infections

mNGS has demonstrated exceptional diagnostic power in rare or culture-negative CNS infections. It has successfully detected *Brucella* spp. [48,49], *Taenia solium* [50,51], *Naegleria fowleri* [52], *Vibrio vulnificus* [53], and *Toxoplasma gondii* [54] in CSF samples. In neurobrucellosis, mNGS identified 11–104 *Brucella* reads, enabling rapid confirmation. For neurocysticercosis, up to 10^5^ *T. solium* reads were detected, with sequencing reads declining after antiparasitic therapy, indicating utility for treatment monitoring. The first Chinese cases of *N. fowleri* and *V. vulnificus* CNS infections were rapidly diagnosed by mNGS, while 65,357 *T. gondii*-specific reads confirmed toxoplasmic encephalitis in an immunosuppressed host.

#### 3.1.2. Application of tNGS in Central Nervous System Infections

##### Clinical Value and Advantages of tNGS in CNS Infections

Unlike mNGS, which sequences all nucleic acids in a sample, tNGS enriches and amplifies specific microbial genomic regions through multiplex PCR or probe hybridization, allowing the deep coverage of predefined loci. This design confers higher analytical sensitivity, lower background interference, and faster turnaround time—features particularly advantageous for low-biomass samples such as CSF. A comparative study of 38 meningitis patients demonstrated that tNGS achieved a higher sensitivity (70.8%) than mNGS (41.7%), with shorter diagnostic time (15 vs. 24 h) and lower cost [55]. Although the specificity of mNGS was slightly higher (78.6% vs. 64.3%), the overall diagnostic yield and clinical applicability favored tNGS, especially when the causative pathogen was among the covered targets. These findings highlight tNGS as a practical and cost-effective option for rapid pathogen identification in CNS infections, complementing the broader but less sensitive mNGS approach.

##### tNGS in Bacterial CNS Infections

tNGS has shown remarkable potential in detecting rare or fastidious bacterial pathogens that are difficult to culture. For example, a case report described *Listeria* monocytogenes meningitis in a 66-year-old man with diabetes and a history of pituitary surgery [56]. While conventional culture remained negative after antibiotic treatment, mNGS successfully detected L. monocytogenes, and subsequent tNGS confirmed its presence and quantitatively tracked the decline in bacterial copy number during therapy. The patient’s favorable recovery demonstrated that tNGS not only enhances diagnostic sensitivity but also enables dynamic monitoring of pathogen burden, thereby guiding antimicrobial management in CNS infections with low residual bacterial load.

##### tNGS in Viral CNS Infections

tNGS also offers precise detection for neurotropic viruses when applied in conjunction with multiplex PCR or hybrid-capture enrichment. In a case of HSV-1 encephalitis [57], mNGS combined with tNGS detected 188 HSV-1–specific sequences in the CSF, and the viral read ratio significantly decreased after acyclovir and foscarnet therapy. This finding demonstrated that tNGS can be used for quantitative monitoring of viral clearance, improving the evaluation of treatment efficacy and disease progression. Furthermore, a probe-capture-based viral tNGS platform (VirCapSeq-VERT) was applied to 73 adults with community-acquired meningitis and detected unexpected viral agents such as *Toscana virus*, *rotavirus*, and *Saffold virus* [58]. The study showed that tNGS could expand the spectrum of viral detection beyond routine PCR panels, allowing identification of atypical and emerging viral pathogens and reducing unnecessary antimicrobial exposure through precision-directed therapy.

##### tNGS in Fungal and Opportunistic CNS Infections

In immunocompromised hosts, tNGS has proven valuable for diagnosing rare opportunistic infections. A case report described a 47-year-old AIDS patient initially misdiagnosed with *Toxoplasma gondii encephalitis* [59]. While mNGS failed to identify a pathogen, tNGS of CSF successfully detected *Aspergillus fumigatus*, revising the diagnosis to CNS aspergillosis. The patient’s condition improved markedly after targeted antifungal therapy. This case exemplifies tNGS’s ability to detect low-abundance or refractory pathogens that may escape detection by unbiased metagenomic sequencing, especially in cases of low fungal load or post-treatment samples.

##### tNGS in Pediatric and Postoperative CNS Infections

The diagnostic value of tNGS has also been verified in pediatric neurosurgical settings. A retrospective study of 35 children with suspected postoperative CNS infections reported that tNGS exhibited a sensitivity of 81.8%, far exceeding that of traditional CSF culture and smear (13.6%), with an area under the ROC curve (AUC) of 0.794 versus 0.568 [60]. These results indicate that tNGS can reliably detect pathogens and their virulence or resistance genes in postoperative or trauma-related intracranial infections, facilitating early and precise antimicrobial interventions.

### 3.2. Respiratory System Infections (RSI)

#### 3.2.1. Application of mNGS in Respiratory Infections

##### mNGS in Common Respiratory Infections

mNGS has revolutionized the diagnosis of pulmonary infections by overcoming the limitations of traditional culture-based diagnostic methods, such as sputum culture and BALF culture. A large-scale multicenter study involving 2388 patients with severe acute respiratory infections (SARI) demonstrated that mNGS identified pathogens in 76% of cases, significantly improving pathogen detection compared to conventional methods [61]. mNGS showed exceptional sensitivity in detecting a wide range of pathogens, including influenza A, influenza B, and respiratory syncytial virus (RSV), which were often the underlying causes of SARI. Furthermore, mNGS exhibited superior diagnostic yield, particularly in cases of polymicrobial infections commonly encountered in critically ill patients. These findings underscore the potential of mNGS to provide rapid, precise pathogen identification that can directly inform timely treatment decisions.

In a retrospective study on the use of mNGS in diagnosing mixed pulmonary infections, mNGS demonstrated remarkably higher sensitivity, achieving 97.2% sensitivity for mixed infections, vastly outperforming traditional methods that had a sensitivity of just 13.9% [62]. This remarkable sensitivity was particularly beneficial for diagnosing fungal infections, with mNGS detecting pathogens like human cytomegalovirus (HCMV) and *Pneumocystis jirovecii* that were missed by conventional methods. In conclusion, mNGS proves invaluable in the management of complex respiratory infections, especially in immunocompromised patients.

##### mNGS in Viral and Fungal Respiratory Infections

The ability of mNGS to identify both common and rare viral and fungal pathogens in respiratory infections has significantly expanded its clinical utility. In particular, mNGS has shown high efficacy in detecting viral pathogens in immunocompromised patients, including HSV-1 and human herpesvirus type 5 (HHV-5), which were often missed by conventional diagnostic methods [63]. Additionally, in COVID-19 patients, mNGS successfully identified fungal co-infections, such as *Aspergillus*, which are common in critically ill patients and those with compromised immunity [64]. This highlights the power of mNGS to identify concurrent viral, bacterial, and fungal pathogens, providing a comprehensive microbial landscape for clinicians to act upon.

mNGS has also proven particularly useful for distinguishing between infection and colonization, especially in fungal infections like *Aspergillus*. In a study of respiratory infections, mNGS exhibited an AUC of 0.894 for distinguishing between Aspergillus infection and colonization, a task where conventional methods struggled [65]. The ability to differentiate between these two conditions is crucial for determining the appropriate treatment plan, particularly in critically ill patients with mixed infections, where decisions about escalating antifungal therapy can be complex. This capability ensures that clinicians can avoid unnecessary antifungal treatments while promptly addressing true invasive infections.

##### mNGS in Mixed Infections and Clinical Management

One of the key strengths of mNGS is its ability to diagnose mixed infections, where multiple pathogens are involved, often in challenging clinical scenarios. In a study of 62 cancer patients with severe pneumonia, mNGS identified polymicrobial infections in 62.9% of cases, compared to just 12.9% identified by traditional culture methods [66]. This demonstrates the superiority of mNGS in diagnosing complex infections where multiple pathogens are involved, emphasizing its potential for optimizing treatment strategies. The ability to identify multiple pathogens in a single test streamlines the diagnostic process, enabling clinicians to more accurately target therapy and reduce unnecessary broad-spectrum antibiotic use.

In immunocompromised patients, mNGS has proven to be highly sensitive, detecting pathogens in 94.5% of cases that conventional methods had missed. This includes detecting pathogens such as HCMV and *P. jirovecii*, which are often difficult to identify through traditional diagnostic techniques [63]. mNGS plays a pivotal role in guiding clinical management by providing a more accurate and timely diagnosis, which is crucial for improving patient outcomes in complex and severe respiratory infections.

The application of mNGS in diagnosing severe pneumonia, particularly in critically ill patients, has also been shown to reduce mortality rates. In a study conducted in Wuhan, patients who underwent mNGS testing had significantly lower 28-day and 90-day mortality rates compared to those who received conventional microbiological testing [67]. This highlights the role of mNGS in not only improving diagnostic accuracy but also in enhancing clinical outcomes by enabling early and targeted therapy. The reduced mortality demonstrates the clinical value of rapid pathogen identification in guiding appropriate antimicrobial therapy.

Additionally, mNGS plays a vital role in guiding optimized antimicrobial therapy for nosocomial lower respiratory tract infections (nLRTIs), including hospital-acquired pneumonia (HAP) and ventilator-associated pneumonia (VAP). In the context of these infections, mNGS has been shown to improve pathogen detection and reduce the prolonged processing times associated with traditional culture methods. This ability to rapidly identify pathogens is critical for timely decision making, enabling more targeted antimicrobial treatment and mitigating the growing threat of antimicrobial resistance [68].

##### mNGS in Novel Pathogen Detection

mNGS has demonstrated exceptional capability in identifying novel and emerging pathogens in severe respiratory infections. A landmark study highlighted mNGS’s role in identifying a novel coronavirus, closely related to SARS-CoV, in the BALF of a patient with severe pneumonia [67]. In this groundbreaking case, mNGS provided the complete genome sequence of the new coronavirus, later named SARS-CoV-2, highlighting its pivotal role in identifying and tracking emerging pathogens in public health crises [67]. The identification of SARS-like coronaviruses using mNGS on a patient’s BALF further strengthens the role of mNGS in detecting previously unknown pathogens in severe respiratory infections. The novel coronavirus identified in this case shares significant genomic similarities with SARS-CoV, underlining the potential of mNGS in identifying and characterizing novel viruses during pandemics or new outbreaks. As demonstrated by the discovery of the virus, mNGS can identify novel pathogens rapidly and precisely, the capability renders it indispensable for the early detection of emerging infectious diseases and provides critical guidance for the development of targeted therapeutics in outbreak scenarios.

#### 3.2.2. Application of tNGS in Respiratory Infections

##### Comparative Performance of tNGS and mNGS in Respiratory Infections

With the increasing diversity of respiratory infections, traditional culturing methods often fail to meet clinical needs, particularly when pathogens are difficult to culture or when antibiotic treatments have already affected pathogen growth. tNGS and mNGS have emerged as powerful tools for diagnosing respiratory infections. tNGS typically provides a more rapid, accurate, and cost-effective solution through the enrichment and amplification of specific pathogens and AMR markers.

A study evaluating tNGS and mNGS in 202 patients with suspected pulmonary tuberculosis (PTB) showed that tNGS and mNGS had sensitivities of 77.66% and 100%, respectively, with specificity at 100% [69]. tNGS demonstrated greater clinical utility due to its fast processing time and lower costs, while also effectively detecting multiple pathogens [69]. A study comparing tNGS and mNGS in 180 patients with respiratory infections demonstrated that tNGS provided similar performance to mNGS in pathogen detection, with tNGS offering a slightly higher detection rate for certain pathogens [70]. tNGS demonstrated a sensitivity of 94.74%, significantly outperforming conventional microbiological methods (CMTs) with a pathogen detection rate of 60.3% compared to 24.4% for CMTs [71]. Both techniques showed advantages in polymicrobial detection, but the clinical significance of co-detected microorganisms needs careful interpretation to differentiate colonization from active infection. This comparison highlights the complementary roles of tNGS and mNGS in respiratory diagnostics, with tNGS offering a pragmatic balance of speed, cost, and accuracy for targeted pathogen detection.

##### tNGS in Respiratory Pathogen Detection

In diagnosing lower respiratory tract infections, tNGS has been shown to effectively complement traditional microbiological methods, especially when pathogens are difficult to culture. In a study of 229 BALF samples, tNGS detected *Pneumocystis jirovecii*, a fungus not identified by mNGS. Additionally, tNGS detected filamentous fungi like *Rhizopus oryzae* and *Aspergillus niger*, which were missed by other methods [21]. This study highlighted tNGS’s broad-spectrum pathogen detection capability, though some anaerobic bacteria were not detected by tNGS, indicating the need for careful selection of diagnostic platforms based on suspected pathogens.

Moreover, tNGS has proven effective in detecting viral co-infections, such as SARS-CoV-2 and RSV, demonstrating its importance in post-pandemic studies for investigating viral co-circulation and understanding the dynamics of respiratory viral infections [72]. The ability to simultaneously detect multiple viral and bacterial pathogens makes tNGS a valuable tool for comprehensive respiratory infection surveillance and management.

##### tNGS in Antimicrobial Resistance Detection

Despite their similar performance in pathogen detection, tNGS showed slightly higher detection rates for some pathogens compared to mNGS in a cohort of 180 patients [70]. However, due to panel coverage limitations, tNGS identified fewer species than mNGS, though the majority of these were clinically relevant. Both methods improved pathogen identification in 30.6% (tNGS) and 33.9% (mNGS) of cases compared to conventional testing. The study demonstrated that tNGS, while showing slightly less broad coverage than mNGS, remains an efficient and cost-effective diagnostic option, particularly when focused detection of clinically significant pathogens is prioritized.

Beyond pathogen identification, tNGS provides valuable information on antimicrobial resistance genes. In a study on BALF, tNGS identified resistance genes such as those conferring resistance to tetracycline, macrolides, and beta-lactams, helping clinicians make informed decisions about antimicrobial treatment [73]. This capability is particularly important in the context of rising antimicrobial resistance, as it enables rapid adaptation of antibiotic regimens based on the detected resistance profiles, potentially improving treatment outcomes and reducing the selective pressure for resistance development.

##### tNGS in Pulmonary Tuberculosis Diagnosis

tNGS has demonstrated its superiority in diagnosing PTB compared to traditional methods such as acid-fast staining and TB culture. In a study of 202 patients with suspected PTB, tNGS showed higher sensitivity and specificity than conventional methods and detected additional pathogens, providing a more comprehensive diagnosis [69]. The rapid turnaround time of tNGS, typically within 24–48 h compared to several weeks for culture, enables earlier initiation of appropriate therapy and better infection control measures. Furthermore, tNGS can detect drug resistance mutations in *Mycobacterium tuberculosis*, facilitating personalized treatment strategies and reducing the risk of multidrug-resistant tuberculosis development.

### 3.3. Bloodstream Infections (BSI)

#### 3.3.1. Application of mNGS in Bloodstream Infections

##### mNGS in Bloodstream Infections

In a multicenter study, mNGS was used to analyze plasma cell free DNA (cfDNA) extracted from 78 intensive care unit (ICU) patients’ blood samples. The results showed that mNGS successfully identified 15 cases that contain pathogens from 78 patients’ blood samples, while conventional blood cultures only diagnosed 10 cases. Among these 78 samples, 7 cases matched both mNGS and blood culture, indicating that mNGS effectively increased pathogen detection, especially when conventional blood cultures failed to detect pathogens [74]. Additionally, the advantages of mNGS in viral detection were demonstrated, with 14 cases where mNGS successfully detected viral pathogens [74].

Another study involving 511 clinical samples showed that mNGS had a sensitivity of 50.7% and specificity of 85.7% for detecting infectious diseases, and it was able to detect more bacteria, fungi, or viruses [75]. In some culture-negative cases, mNGS showed a higher sensitivity for detecting Mycobacterium tuberculosis [75].

In a study of sepsis, mNGS significantly increased the detection rate of bacterial pathogens. The results showed that mNGS was able to detect more bacteria, such as *Escherichia coli* and *Klebsiella pneumoniae*, in patients where conventional blood cultures failed to detect pathogens. For patients who had received antibiotics, mNGS still provided valuable information, with 46% of culture-negative cases accurately identified by mNGS [76]. Furthermore, mNGS maintained high sensitivity and specificity for bacterial diagnosis in patients who had received antibiotics, outperforming traditional culture methods [76]. This capability is particularly valuable in the clinical setting, where empirical antibiotic therapy is often initiated before diagnostic results are available, rendering subsequent culture-based diagnostics less effective.

Additionally, mNGS proved valuable in identifying co-infections and detecting antimicrobial resistance markers. A study demonstrated that mNGS could detect antibiotic resistance genes, such as beta-lactamase and tetracycline resistance genes, which are often overlooked in clinical settings but are crucial for treating septic patients [77]. The ability to simultaneously identify pathogens and their resistance profiles enables more targeted antimicrobial therapy, reduces the use of broad-spectrum antibiotics, and may help mitigate the development of antimicrobial resistance.

mNGS also demonstrated significant advantages in detecting viral bloodstream infections. For example, in studies of hepatitis viruses, cytomegalovirus (CMV), and HSV infections, mNGS successfully detected viral pathogens that conventional methods could not identify [78]. In a special case involving a lung transplant patient, mNGS detected an undiagnosed viral infection by analyzing cfDNA, which was not identified by conventional tests [78]. This demonstrates the utility of mNGS in detecting low-abundance viral pathogens and provides a noninvasive diagnostic approach for monitoring infection in immunocompromised patients.

Despite its potential in viral detection, mNGS still faces challenges, such as delayed sample collection, low viral titers, and limitations in RNA sequencing coverage. Thus, the combined use of DNA and RNA sequencing will be crucial for improving viral detection accuracy [38]. Future technological advances in enrichment methods and sequencing protocols specifically designed for viral detection may help overcome these limitations and expand the clinical utility of mNGS in diagnosing viral bloodstream infections.

Fungal infections, especially those caused by *Candida* and *Aspergillus* species, are often difficult to diagnose and treat, particularly in immunocompromised patients. mNGS has made significant progress in this area. For example, mNGS can detect Candida species and other fungi in cases where traditional culture methods cannot quickly identify pathogens [75]. A study on severe sepsis patients showed that mNGS could detect fungal infections early and provide rapid pathogen information for clinicians [75]. Early detection of fungal pathogens is critical, as delayed diagnosis and treatment of invasive fungal infections are associated with high mortality rates. The ability of mNGS to provide rapid results enables earlier initiation of appropriate antifungal therapy, potentially improving patient outcomes.

These findings demonstrate that mNGS has been proven to effectively detect pathogens in bloodstream infections, particularly when traditional methods are unable to identify them, offering a valuable diagnostic alternative in challenging clinical scenarios.

##### mNGS in Transfusion-Related Sepsis

Transfusion-related sepsis remains an important challenge in hospital infection control. Traditional bacterial culture methods often fail to detect pathogens in a timely manner, especially in cases of antibiotic pre-treatment. mNGS has demonstrated higher precision in pathogen detection in transfusion-related sepsis cases. In a study involving three cases of Gram-negative bacterial transfusion-related sepsis, mNGS successfully detected the pathogens and discovered a novel *Acinetobacter* species in a platelet product, despite photochemical pathogen reduction treatment [79]. This highlights the potential of mNGS not only for diagnosing transfusion-related infections but also for quality control and safety monitoring of blood products, potentially preventing transmission of infectious agents through transfusion.

##### mNGS in Cell-Free DNA Detection

cfDNA sequencing provides a noninvasive method for detecting infections across the body. Studies have shown that mNGS, when applied to cfDNA from 1250 sepsis alert patients demonstrated high sensitivity and specificity towards bacteria, DNA viruses, fungi, and parasites. In a study of 350 sepsis alert patients, mNGS achieved a diagnostic accuracy of 93.7%, identifying the pathogen more frequently than conventional blood cultures [80]. The use of cfDNA as a sample type offers several advantages, including the ability to detect pathogens from multiple body sites, reduced invasiveness compared to tissue biopsies, and the potential for serial monitoring of infection dynamics and treatment response. This approach is particularly valuable in critically ill patients where repeated invasive sampling may not be feasible or safe.

##### Clinical Impact of mNGS in Routine Practice

A retrospective cohort study evaluated the clinical impact of mNGS (Karius test) in 82 patients across five U.S. institutions. The positivity rate was 61%, with significant results observed primarily for bacterial and fungal pathogens. However, the clinical impact of mNGS in routine practice was limited, with only 7.3% of cases having a positive impact [81]. This finding highlights an important distinction between diagnostic yield and clinical utility. While mNGS may detect pathogens, its clinical impact depends on several factors, including the clinical context, timing of testing, availability of alternative diagnostics, and the actionability of results. These observations underscore the need for careful patient selection, appropriate clinical indications for mNGS testing, and integration of results with clinical judgment to maximize its clinical value.

#### 3.3.2. Application of tNGS in Bloodstream Infections

##### Comparative Performance of tNGS and mNGS in Bloodstream Infections

Bloodstream infections (BSIs) are a major cause of morbidity and mortality worldwide. Traditional diagnostic methods such as blood cultures are time-consuming and often fail to detect pathogens, especially in cases where antibiotics have already been administered or when pathogens are difficult to grow in culture. NGS technologies, particularly tNGS and mNGS, have shown promising potential for improving pathogen detection in BSIs.

Both tNGS and mNGS showed higher efficiency in detecting a wide range of pathogens, including bacteria, fungi, and viruses, although tNGS was faster and more cost-effective [82]. These findings suggest that tNGS represents a practical alternative to mNGS for bloodstream infection diagnostics, offering comparable diagnostic performance with improved turnaround time and reduced costs, making it more suitable for routine clinical implementation.

##### tNGS in Pathogen Detection in Bloodstream Infections

tNGS has been proven effective in detecting a broad spectrum of pathogens in bloodstream infections, especially when conventional methods fail. In a study using a multiplex tNGS panel targeting over 330 clinically relevant pathogens, tNGS significantly increased pathogen detection, with a 6- to 8-fold increase in pathogen reads compared to traditional blood culture methods [83]. This is particularly useful in diagnosing infections caused by low-abundance pathogens or those that are difficult to culture. Additionally, tNGS has shown high sensitivity for detecting opportunistic pathogens, including those not typically covered by routine microbiological tests [83]. The enrichment strategies employed by tNGS overcome the challenge of low pathogen-to-host DNA ratios in blood samples, which often limit the sensitivity of unbiased metagenomic approaches.

One notable application of tNGS and mNGS is their ability to detect rare or mixed infections. For example, in a patient with co-infection of *Orientia tsutsugamushi* and influenza A virus, tNGS helped identify both pathogens simultaneously, providing valuable information for clinical management and treatment [84]. The ability to detect multiple pathogens in a single assay is particularly important in immunocompromised patients and those with complex clinical presentations, where polymicrobial infections are more common and may require tailored therapeutic approaches.

##### tNGS in Antimicrobial Resistance Detection

In addition to pathogen identification, tNGS can also detect AMR genes, which is crucial for guiding appropriate treatment strategies. A study on bloodstream infections demonstrated that tNGS was able to identify key resistance markers, including those for tetracycline, macrolides, and beta-lactams [73]. This ability to identify AMR genes directly from blood samples enables faster adjustments, which is crucial; inappropriate antibiotic therapy is associated with increased mortality.

Furthermore, tNGS has shown its potential in detecting drug resistance in pathogens that are not easily cultured or are resistant to conventional antibiotics. This capability significantly improves treatment outcomes by enabling clinicians to tailor antibiotic therapy based on the specific resistance profiles of the pathogens detected [85]. The integration of resistance gene detection with pathogen identification in a single test streamlines the diagnostic process and provides actionable information for antimicrobial stewardship programs, potentially reducing the inappropriate use of broad-spectrum antibiotics and helping to combat the growing threat of antimicrobial resistance.

##### tNGS in the Diagnosis of Rare and Complex Infections

tNGS is particularly useful in diagnosing rare or complex infections that may be overlooked by traditional methods. For instance, in a case of disseminated *Mycobacterium bovis* infection in an immunocompromised child, tNGS successfully identified the pathogen, which was missed by conventional tests [86]. This highlights the role of tNGS in diagnosing atypical infections and its importance in providing a comprehensive pathogen profile, especially in immunocompromised patients where opportunistic infections are more common and may present with atypical clinical features.

Additionally, tNGS has been used to detect *Toxoplasma gondii* in blood and CSF in a pediatric leukemia patient who developed toxoplasmic encephalitis [87]. This case emphasizes the utility of tNGS in detecting rare pathogens in immunocompromised patients, facilitating early diagnosis and timely treatment. The ability to rapidly identify such pathogens is critical, as delayed diagnosis and treatment of these infections can lead to severe complications and poor outcomes. tNGS provides a valuable diagnostic tool for these challenging cases where traditional methods may have insufficient sensitivity or prolonged turnaround times.

##### Advantages of tNGS over Traditional Diagnostic Methods

tNGS offers several advantages over traditional diagnostic techniques, particularly in its ability to provide rapid, comprehensive pathogen detection. Compared to conventional blood cultures, tNGS can detect a wider range of pathogens, including those that are difficult to culture or those that cause mixed infections. For example, in the study of *Mycoplasma pneumoniae* and respiratory infections, tNGS successfully identified pathogens that were missed by culture methods and provided a broader pathogen spectrum, including viral co-infections [88]. The enhanced detection capability of tNGS is particularly valuable in cases where patients have received empirical antibiotic therapy, which may render subsequent cultures negative while the patient remains clinically unstable.

Furthermore, tNGS is more cost-effective and time-efficient than mNGS, making it more feasible for routine clinical use. A study combining tNGS with a human cell-specific filtration membrane showed that tNGS not only improved pathogen detection sensitivity but also reduced the background interference from host DNA, achieving a significant increase in pathogen reads [83]. This highlights the potential of tNGS to be integrated into clinical workflows for rapid and accurate diagnosis. The combination of improved sensitivity, rapid turnaround, and cost-effectiveness positions tNGS as a promising tool for routine bloodstream infection diagnostics, potentially replacing or complementing traditional culture-based methods in many clinical scenarios.

### 3.4. Digestive System Infections

#### 3.4.1. Application of mNGS in Digestive System Infections

##### mNGS in Parasitic Digestive Infections

Parasitic infections pose significant diagnostic challenges due to limitations of stool microscopy and culture. mNGS has demonstrated exceptional performance in detecting *Cryptosporidium* in patients with severe diarrhea, showing high sensitivity and specificity particularly in immunocompromised hosts at elevated risk for atypical presentations [89]. In a 47-year-old male with *Enterocytozoon bieneusi* infection, mNGS successfully identified the pathogen missed by conventional diagnostics, enabling targeted therapy and complete resolution [90]. Similarly, in a 10-year-old child with severe cryptosporidiosis, mNGS provided timely diagnosis after conventional methods failed, facilitating appropriate treatment and recovery [89]. These cases highlight mNGS’s superior detection capability for parasites that evade traditional microscopy or present with atypical features.

##### mNGS in Fungal Digestive Infections

Opportunistic fungal pathogens frequently complicate immunocompromised states, often presenting diagnostic difficulties. In a renal transplant recipient, mNGS identified *Talaromyces marneffei* in blood and bronchoalveolar lavage fluid, prompting timely voriconazole therapy and favorable outcome [91]. mNGS also successfully diagnosed Histoplasma capsulatum infection, leading to prompt antifungal treatment and recovery [92]. These findings underscore mNGS’s advantages in identifying low-abundance fungal pathogens in complex clinical presentations, particularly *Aspergillus* fumigatus and other opportunistic fungi difficult to culture or frequently misdiagnosed. The ability to detect these pathogens early is crucial for reducing morbidity and mortality in immunosuppressed populations.

##### mNGS in Bacterial and Viral Digestive Infections

Bacterial pathogens in the digestive system, particularly fastidious or anaerobic organisms, challenge conventional culture methods. mNGS detected *Prevotella oris*—typically a benign commensal—as the causative agent of severe pneumonia in a 56-year-old woman after cultures failed [93]. In disseminated *nocardiosis*, mNGS identified *Nocardia farcinica*, a rare pathogen often misdiagnosed as tuberculosis, enabling species-specific therapy and improved outcomes [94]. For viral infections, mNGS identified the causative *bunyavirus* in severe fever with thrombocytopenia syndrome presenting with fever and diarrhea, enabling accurate diagnosis and treatment [94]. In patients with decompensated cirrhosis, mNGS detected non-hepatotropic viruses including cytomegalovirus, which correlated with poorer clinical outcomes, emphasizing the importance of early viral detection in hepatic disease [95].

##### mNGS in Mixed Infections and Resistance Profiling

In rectal swabs from patients harboring multidrug-resistant organisms, mNGS identified resistance genes and pathogens undetected by conventional methods, providing comprehensive microbial landscape assessment [96]. The technology’s ability to rapidly identify antibiotic-resistant bacteria facilitates targeted therapy in severe gastrointestinal infections, particularly valuable in hospitalized and immunocompromised patients where inappropriate antibiotic use correlates with poor outcomes.

### 3.5. Urinary Tract Infections (UTIs)

#### 3.5.1. Application of mNGS in Urinary Tract Infections

##### Clinical Performance of mNGS in UTI Diagnosis

Urinary tract infections represent one of the most common clinical infections, yet culture-based diagnosis suffers from delayed results and limited detection of mixed or low-abundance pathogens. In 213 urine samples from suspected UTI patients, mNGS demonstrated 81.4% sensitivity and 92.3% specificity compared to routine culture, with overall accuracy of 84.4%. When assessed against composite diagnostic standards (Sensitivity and specificity of mNGS were calculated using two criteria: (1) a gold standard based on clinical culture for fungi and bacteria; (2) a composite standard based on a combination of clinical testing (culture, microscopy, and pathology), confirmatory qPCR testing, and clinical adjudication by doctor), performance improved to 89.9% sensitivity and 100% specificity [97]. To avoid false-positive results from the low-level DNA background of the reagents, microbial contamination, and urethral colonizing flora, threshold criteria were established for pathogen detection: (1) the threshold should be above the optimal normalized reads per million (nRPM) threshold; (2) the threshold should be above the lowest-read number threshold of target pathogen; (3) the threshold should exclude the non-pathogenic species or probiotics [97]. Comparative analysis revealed mNGS detected at least one pathogen in 87.9% of UTI cases versus 30.3% by culture, identifying 26 species compared to only 5 species by culture (the results of culture and mNGS detection were interpreted by at least 3 urology, infectious disease and microbiology specialists, taking into account the patient’s clinical background) [98]. The sensitivity and specificity of mNGS for the diagnosis of UTI, were 100% and 50% [98]. Notably, mNGS detected fastidious organisms including anaerobic bacteria and Mycobacterium tuberculosis—pathogens routinely missed by traditional methods.

##### mNGS in Mixed and Recurrent UTIs

mNGS demonstrates superior performance in detecting polymicrobial infections frequently missed by culture. In 19 kidney transplant recipients (KTRs) with recurrent UTIs (RUTIs), mNGS detected mixed infections in 89.5% of cases versus 10.5% by culture, additionally identifying viruses and fungi not captured by routine methods [99]. Comparative analysis in KTRs showed mNGS outperformed culture across bacterial, viral, and fungal detection, leading to modifications in anti-infection therapy in 76.9% of cases and significantly improving patient outcomes [99]. In special populations including 50 cutaneous ureterostomy (CU) patients, mNGS achieved 91.4% sensitivity and 76.3% specificity, detecting broader pathogen diversity and identifying biomarkers including *Citrobacter freundii* and *Klebsiella oxytoca* associated with increased inflammatory responses [100].

##### mNGS in Atypical and Opportunistic UTIs

Viral and fungal UTIs pose particular diagnostic challenges in immunocompromised hosts. mNGS successfully identified *Orientia tsutsugamushi* in a patient initially presenting with UTI symptoms (not a primary UTI) and misdiagnosed with influenza; subsequent doxycycline therapy based on mNGS results led to clinical improvement [101]. In KTRs with recurrent UTIs, mNGS detected fungal pathogens including *Candida auris* missed by culture, proving crucial for managing opportunistic infections in high-risk populations [99]. Studies exploring urinary viruses in overactive bladder patients revealed John Cunningham virus as the most frequently detected pathogen, suggesting viral infections may exacerbate symptoms and alter bacterial urobiome composition [102]. These findings highlight mNGS’s capacity to detect atypical pathogens with overlapping clinical presentations, preventing misdiagnosis and treatment delays.

##### Appropriate Indications for mNGS in UTIs

In conclusion, appropriate indications for mNGS in UTIs include: (1) recurrent or complicated UTIs with negative cultures despite high clinical suspicion, particularly in patients with prior antibiotic exposure; (2) immunocompromised patients (e.g., kidney transplant recipients, neutropenic patients) where opportunistic or atypical pathogens are suspected; (3) suspected polymicrobial infections involving fastidious, anaerobic, or non-culturable organisms; (4) severe or life-threatening urinary tract infections requiring rapid etiological identification when conventional tests remain negative; (5) epidemiological or infection control investigations requiring comprehensive pathogen and resistance gene profiling. In these selected populations, the additional diagnostic yield of mNGS can provide critical information that directly influences patient management and justifies its higher cost.

#### 3.5.2. NGS in Antimicrobial Resistance Detection

The rising prevalence of multidrug-resistant (MDR) organisms in UTIs, including Carbapenem-Resistant Enterobacteriaceae (CRE), Extended-Spectrum Beta-Lactamase (ESBL) *E. coli*, and vancomycin-resistant enterococci (VRE), presents a growing therapeutic challenge [103,104]. Conventional urine culture with antimicrobial susceptibility testing (AST) typically requires 48–72 h for complete results, potentially delaying optimal therapy in patients with resistant infections. Both mNGS and tNGS offer the capability to simultaneously detect pathogens and identify ARGs directly from urine specimens, providing actionable information days earlier than traditional methods.

Unlike PCR, the mNGS approach is target-agnostic and does not require prior microorganism knowledge [105]. The whole-genome shotgun sequencing of urine samples enables comprehensive profiling of resistance determinants without prior knowledge of the pathogen. Studies have demonstrated that mNGS can detect a wide range of resistance organisms precisely in urine, including *Escherichia coli*, *Klebsiella pneumoniae*, *Staphylococcus aureus*, *Candida auris* [100]. The semi-quantitative nature of mNGS also enables the assessment of resistance gene abundance, which may help differentiate between dominant resistant pathogens and low-abundance resistance determinants carried by commensal organisms.

tNGS offers a more targeted approach for AMR detection in UTIs, with panels specifically designed to capture clinically relevant resistance genes alongside pathogen identification. In a multicenter cohort study focused on preoperative infection in urolithiasis, both mNGS and tNGS methods maintained 100% concordance with culture-positive results in clear and turbid urine samples, with tNGS demonstrating fewer false positives [106]. For antibiotic resistance prediction, tNGS detected more ARGs (52.67% vs. 41.22% for mNGS) and achieved 100% sensitivity for vancomycin and methicillin resistance in Gram-positive pathogens [107]. However, tNGS is also limited to detecting organisms included in its predetermined panel.

The clinical relevance of NGS-based AMR detection in UTIs extends beyond individual patient management. However, several challenges remain in the routine implementation of NGS-based AMR detection for UTIs. The correlation between genotypic resistance (detected resistance genes) and phenotypic resistance (expressed in clinical isolates) requires careful interpretation, as the presence of a resistance gene does not always guarantee phenotypic resistance, and resistance may be mediated by mechanisms not captured by current panels or databases. Additionally, standardization of bioinformatics pipelines for resistance gene identification and reporting, as well as establishment of clinically validated interpretation criteria, remain areas requiring further development.

## 4. Application of Metagenomics in Healthcare-Associated Infection and Antimicrobial Resistance Surveillance

### 4.1. The Hospital Wastewater Resistome

Multiple studies have identified hospital wastewater as a significant reservoir and diffusion channel for ARGs. Metagenomic analyses have consistently shown that hospital effluents have a greater resistance abundance and diversity than wastewater treatment plant (WWTP) and community influents [108]. This enriched resistome has been shown to reflect within-hospital clinical activity and outbreaks [109,110,111]. The greater abundance and diversity of ARGs in hospital wastewater can be explained by the high antibiotic selective pressure and the unique patient population. Hospitalization and longer hospital stays are known risk factors for acquiring resistance genes [112,113]. Furthermore, hospital wastewater contains greater concentrations of antibiotics, antiseptics, and other drugs than municipal wastewater, creating a potent environment for the selection of resistant bacteria [114,115,116].

### 4.2. Comparison of Hospital and Community Resistomes

Despite the high enrichment of ARGs in hospital wastewater, its signature in downstream WWTP influent is often weak. Studies comparing the resistance profile of hospital effluent and WWTP influent have generally concluded that hospitals make only a small or no contribution to downstream resistance [117,118,119]. The WWTP resistome is typically more similar to the community effluent resistome, suggesting that WWTP samples represent the community resistome more closely [117,118]. This is likely due to the significant dilution of hospital wastewater with domestic effluents, with estimates suggesting hospital wastewater may constitute only about 1% of the total volume in urban catchment areas [117,119]. However, positive associations between resistance genes in hospital effluent and WWTP sewage have been observed, indicating a detectable, albeit diluted, link [120,121].

### 4.3. Environmental Dissemination of Hospital-Associated Resistant Bacteria

Genome-resolved metagenomics has provided high-resolution evidence for the transmission of hospital-adapted clones into the urban environment. A notable study traced a multidrug-resistant *Klebsiella pneumoniae* ST-11 clone, responsible for a 9-month hospital outbreak, to urban waters miles away from the hospital [122]. The abundance of this pathogen and its associated carbapenem-resistance megaplasmid, pKPC-146, significantly increased in the environment concurrently with the hospital outbreak, demonstrating a direct impact of hospital-derived fecal wastes on the urban microbiome. Furthermore, the geospatial dissemination of such resistant plasmids has been shown to inversely correlate with the quality of sewage infrastructure, highlighting the role of the built environment in public health exposure to resistant pathogens [122].

## 5. Summary and Outlook

### 5.1. Overall Assessment of NGS Technologies in Infectious Disease Diagnostics

#### 5.1.1. Clinical Value and Application Positioning of mNGS

mNGS has emerged as a revolutionary diagnostic tool in the clinical diagnosis of infectious diseases, demonstrating significant advantages. Its core value lies in providing rapid, unbiased, and highly sensitive pathogen detection methods capable of simultaneously identifying multiple pathogen types including bacteria, viruses, fungi, and parasites in a single test. Multiple clinical studies have demonstrated clear diagnostic advantages of mNGS over traditional culture methods (Table 2). A study involving 109 adult patients showed that mNGS exhibited significantly higher pathogen detection rates than traditional culture in blood, BALF, and sputum samples (sensitivity 67.4% vs. 23.6%), with comparable specificity. Notably, mNGS positivity correlated with longer hospital stays and higher 28-day mortality, suggesting that early mNGS application can help identify high-risk patients and optimize infection management [123]. In a cohort of 76 patients with suspected spinal infections, mNGS demonstrated markedly higher detection rates (77.6% vs. 18.4%) and sensitivity (82.3% vs. 17.5%) than conventional culture, with shorter diagnostic turnaround time (1.65 vs. 3.07 days) [124].

The principal technical advantages of mNGS include its comprehensive and unbiased approach, as it does not require prior hypotheses about which pathogens might be present and allows for simultaneous detection and identification of a wide array of pathogens from a single sample, including rare pathogens, those presenting atypically, or those for which no targeted diagnostics exist [125]. In terms of rapidity, compared to traditional culture methods that typically require 48–72 h to obtain results (and even more than a week for fastidious bacteria such as mycobacteria) [7], the average turnaround time for conventional mNGS is 24 h [126], nanopore platform technology can achieve detection times as short as 6 h in dedicated and well organized contexts [7,15]. Additionally, mNGS remains highly effective in situations where patients have already been treated with antibiotics, which can inhibit pathogen growth in cultures and lead to false negative results despite ongoing infection [127]. The technology also provides antimicrobial resistance gene detection capabilities, offering critical information for guiding treatment decisions, and its semiquantitative capabilities allow dynamic monitoring of disease progression to support precision treatment adjustments.

In conclusion, across different infection types (e.g., respiratory, blood, CSF), mNGS has demonstrated broad application value due to several transversal advantages that are not confined to any specific tissue. These include: (1) unbiased pathogen detection with diagnostic accuracy less affected by prior antibiotic use; (2) enhanced sensitivity for mixed, polymicrobial, or culture-negative infections, enabling identification of novel, rare, or fastidious pathogens; (3) comprehensive genomic analysis for antimicrobial resistance gene detection and host response profiling; and (4) versatility across sample types, including noninvasive options such as cfDNA analysis.

#### 5.1.2. Clinical Value and Application Positioning of tNGS

tNGS represents a precise and efficient pathogen detection method playing an increasingly important role in clinical diagnostics. By concentrating sequencing resources on clinically relevant pathogens, tNGS demonstrates clear advantages over traditional methods and untargeted sequencing in detection sensitivity, specificity, cost-effectiveness, and turnaround time.

For clinicians interpreting NGS results, two key technical parameters—sequencing depth and genomic coverage—are essential for understanding test performance and limitations. Sequencing depth refers to the number of times a specific nucleotide position is sequenced and represented by individual reads. Higher depth increases the probability of detecting low-abundance pathogens, particularly in samples where microbial nucleic acids are scarce relative to host background. For clinical sensitivity, adequate depth is crucial because pathogens present at very low concentrations—such as in early infection, after antibiotic treatment, or in paucibacillary specimens like CSF—may be missed entirely if sequencing depth is insufficient. Genomic coverage describes the proportion of a pathogen’s genome that has been successfully sequenced and mapped. Coverage uniformity is equally important; uneven coverage with gaps can lead to false-negative results for specific genomic regions containing resistance determinants or species-specific markers. Together, depth and coverage directly impact clinical utility. Adequate depth ensures detection sensitivity, while sufficient coverage enables accurate pathogen discrimination—distinguishing between closely related species, identifying mixed infections, and detecting antimicrobial resistance mechanisms. Understanding these parameters helps clinicians assess result reliability and recognize when negative results may reflect technical limitations rather than true absence of infection. The core technical advantages of tNGS include targeted enrichment and deep coverage, achieving deeper sequencing depth and more uniform coverage for predefined pathogen targets, thereby improving detection sensitivity for low-abundance pathogens. Compared to mNGS, tNGS offers cost-effectiveness advantages with lower costs, making it more suitable for routine clinical application. The technology provides more reliable pathogen quantification information, helping distinguish colonization from infection to guide treatment decisions, and its simplified operational processes and relatively lower bioinformatics analysis requirements make it more accessible for implementation in primary healthcare facilities.

Across different clinical scenarios, tNGS has demonstrated specific advantages. Similar to mNGS, tNGS demonstrates higher sensitivity than conventional methods across various types of infections (Table 3). In some contexts, its sensitivity is even higher than that of mNGS (Table 3). In CNS infections, compared to mNGS, tNGS provides deeper coverage, and lower cost; it is particularly suitable for cases with known or limited pathogen spectra such as bacterial meningitis, herpes simplex virus (HSV) encephalitis, or postoperative infections. For respiratory infections, tNGS enables rapid, accurate, and cost-effective detection of multiple respiratory pathogens, particularly suitable for guiding empirical therapy and antimicrobial stewardship, with significant advantages in detecting antimicrobial resistance and identifying polymicrobial infections. In bloodstream infections, tNGS provides rapid, accurate, and cost-effective detection of multiple pathogens while simultaneously providing antimicrobial resistance information, obtaining actionable information in a single assay to guide targeted antimicrobial therapy, particularly suitable for critical care settings.

#### 5.1.3. Complementary Relationship Between mNGS and tNGS

mNGS and tNGS should not be viewed as competing technologies but rather as complementary tools that leverage their respective advantages within clinical diagnostic workflows. For a more direct comparison of the respective advantages of mNGS and tNGS, we constructed Table 4. Due to differences in study populations and the pathogens detected, the sensitivities of mNGS and tNGS vary across different scenarios. tNGS is suitable for infections with known or limited pathogen spectra, situations requiring rapid results to guide immediate treatment decisions, routine screening of common pathogens, antimicrobial resistance surveillance, and cost-sensitive clinical environments. In contrast, mNGS is appropriate for complex infections of unknown etiology, atypical infections in immunocompromised patients, suspected rare or emerging pathogen infections, mixed or polymicrobial infections, and cases with negative tNGS results but high clinical suspicion of infection. Clinical practice should adopt a tiered diagnostic strategy with tNGS as a first-line rapid diagnostic tool and mNGS reserved for complex or undiagnosed cases. This complementary application model can significantly improve etiologic diagnostic yield, optimize therapeutic precision, and improve patient prognosis.

### 5.2. Current Major Challenges

#### 5.2.1. Limitations of mNGS

Despite its transformative potential, mNGS faces several limitations. False positives remain a significant issue, often resulting from contamination, species misclassification, or the presence of background microbial DNA, with studies showing that background microorganisms contribute to up to 49% of false positive cases [128], while discrepancies in result interpretation by laboratory personnel can also lead to false positives or missed pathogen identification [129]. To avoid personnel interpretation, it is important for microbiologists and physicians, etc., to review the results before the results are released to the patient. False negatives primarily arise from loss of microbial nucleic acids during the laboratory phase, potentially caused by inefficient extraction, host DNA interference, or excessive fragmentation [130]. Standard mNGS workflows demonstrate lower sensitivity for RNA viruses, and the technology cannot detect infections compartmentalized to specific anatomical sites or pathogens diagnosable only through serology. At present, NGS is largely qualitative method, and it is still a considerable way off from being developed into a reliable quantitative or semi-quantitative tool. For viral loads, qPCR is still the gold standard which can achieve absolute quantification while mNGS only has semi-quantitative capabilities. Therefore, mNGS is recommended for pathogen discovery rather than precise viral load monitoring. High costs remain prohibitive, with second-generation sequencing potentially costing up to USD 500 per sample and third-generation sequencing platforms being even more expensive [131]. While the typical turnaround time for conventional mNGS is approximately 24 h, this timeframe often reflects the cumulative duration of library preparation, sequencing, and bioinformatics analysis. However, in clinical practice, many laboratories adopt batch sequencing workflows to reduce per-sample costs and increase throughput. In such settings, samples are accumulated until a sufficient number is reached to load a sequencing run, which can introduce pre-analytical delays and extend the overall turnaround time beyond the theoretical minimum. Conversely, single-sample or “stat” sequencing protocols—though logistically more costly and resource-intensive—enable immediate processing and are particularly valuable in critical care settings where rapid pathogen identification is essential for guiding timely therapeutic decisions. Furthermore, mNGS workflows have yet to receive approval as in vitro diagnostic (IVD) assays, being mostly used as laboratory-developed tests (LDTs) [132], lacking standardized operating procedures (SOPs). Used as LDTs, the implementation of NGS requires specialized personnel, as well as greater financial and time investment and so on. These barriers collectively contribute to diagnostic inequity, where patients in large academic centers benefit from advanced NGS diagnostics while those in smaller community hospitals remain dependent on conventional methods with well-recognized limitations.

#### 5.2.2. Limitations of tNGS

tNGS similarly faces several limitations in clinical implementation. Detection capability is fundamentally constrained to pathogens within predefined panels, creating diagnostic blind spots when encountering pathogens outside the panel, and in situations involving emerging pathogens, rare infections, or uncharacterized organisms, mNGS retains distinct advantages [1,133,134]. Detection performance depends heavily on reference database quality: sequence errors, inaccurate annotations, or outdated taxonomic classifications can lead to pathogen misidentification. For pathogens with high genetic variability (particularly RNA viruses), sequence divergence between primers/probes and circulating strains may compromise detection sensitivity. Meanwhile, mNGS also faces such limitations. PCR-based multiplex tNGS inherently exhibits amplification biases, with primer design variations, unequal amplification efficiencies, and GC content effects potentially introducing quantitative inaccuracies; meanwhile, in multiplex systems, primer interactions and competitive amplification may affect detection performance for certain targets. Similar to other nucleic acid detection methods, tNGS cannot inherently distinguish between colonizing microbiota and causative pathogens, requiring clinical interpretation to integrate detection results with patient clinical presentation, imaging findings, and pathogen quantification data. As a relatively emerging technology, tNGS standardization remains incomplete regarding detection protocols, result interpretation criteria, and clinical reporting formats, requiring the establishment of result comparability across different panels and platforms through large-scale clinical validation studies and consensus guidelines. The operational complexity from panel design through bioinformatics analysis requires personnel with specialized expertise, potentially limiting implementation in resource-limited settings. Most tNGS assays currently function as LDTs rather than approved IVD devices, similar to mNGS platforms [132].

### 5.3. Future Development Directions

#### 5.3.1. Technical Optimization and Innovation

Future development should focus on cost reduction, as advancing sequencing technology and scaled application promise continued cost decreases to improve clinical accessibility. For mNGS, improving RNA virus detection through combined DNA/RNA sequencing should be prioritized to enhance viral detection sensitivity. For tNGS, pathogen panel coverage should be continuously expanded to encompass emerging pathogens while maintaining cost-effectiveness. Workflow optimization should simplify sample processing, reduce turnaround times, and improve laboratory operational efficiency. Integration of new technology platforms, such as exploring nanopore sequencing applications in clinical diagnostics, could enable faster point-of-care testing.

#### 5.3.2. Standardization and Quality Control

Establishing unified standards is essential, including developing standardized operating procedures for NGS pathogen detection covering all aspects from sample collection, processing, sequencing, and data analysis. Perfecting quality control systems requires developing standard reference materials and proficiency testing programs to ensure inter-laboratory result consistency and comparability. Standardizing result interpretation necessitates establishing standardized clinical reporting formats and interpretation guidelines, including pathogen positivity criteria and clinical significance determination rules. Advancing regulatory certification should promote NGS testing transition from LDT to IVD status, improving regulatory frameworks and approval processes.

#### 5.3.3. Clinical Application Strategy Optimization

Developing tiered diagnostic strategies requires rational selection of mNGS, tNGS, or traditional methods based on clinical scenarios, infection types, and patient characteristics to achieve optimal diagnostic benefits. Integrating clinical decision support systems involves developing intelligent result interpretation and treatment recommendation systems to assist clinicians in diagnostic and therapeutic decision making. Conducting prospective studies through large-scale prospective clinical trials is necessary to evaluate actual NGS impact on patient outcomes and healthcare costs, clarifying optimal application scenarios. Advancing antimicrobial stewardship should leverage NGS rapid pathogen identification and resistance detection capabilities to optimize antimicrobial use and reduce unnecessary broad-spectrum antibiotic application.

#### 5.3.4. Bioinformatics and Artificial Intelligence

Optimizing analytical algorithms requires developing more precise pathogen identification algorithms to reduce false positives and false negatives. Building comprehensive databases necessitates continuous updating and improvement of pathogen genome databases to enhance recognition capabilities for rare and emerging pathogens. Applying machine learning should utilize artificial intelligence technology to assist data interpretation, distinguish colonization from infection, and predict disease severity and prognosis. Integrating multi-omics data should combine genomics, transcriptomics, and host immune response analysis to provide more comprehensive diagnostic information.

#### 5.3.5. Clinical Implementation and Training

Strengthening talent development requires training professionals with comprehensive knowledge in clinical microbiology and molecular biology to improve result interpretation capabilities. Promoting primary-level applications should simplify operational procedures and develop NGS testing protocols suitable for primary healthcare facilities. Establishing multidisciplinary collaboration should promote close cooperation among clinicians, laboratory physicians, bioinformatics experts, and industry to jointly advance clinical translation of technology.

### 5.4. Conclusions

Next-generation sequencing technologies, encompassing both metagenomic (mNGS) and targeted (tNGS) approaches, are fundamentally reshaping the diagnostic landscape for infectious diseases. mNGS offers an unbiased, broad-spectrum detection capability, proving indispensable for diagnosing complex infections of unknown etiology, opportunistic infections in immunocompromised hosts, and emerging pathogen threats. In parallel, tNGS provides a highly sensitive, rapid, and cost-effective solution for the precise identification of common pathogens and is a powerful tool for antimicrobial stewardship. These technologies are not mutually exclusive but are highly complementary; their synergistic application within clinical diagnostic workflows is key to maximizing diagnostic yield and patient benefit. The utility of NGS extends beyond direct patient sampling to innovative public health surveillance. As demonstrated in the context of healthcare-associated infections (HAIs) and AMR, mNGS analysis of hospital wastewater provides a noninvasive means to reveal the distinct and enriched resistome of hospital settings, track the dissemination of outbreak-causing clones into the environment, and monitor the efficacy of infection control measures. This application underscores the potential of NGS to inform a “One Health” approach to combating AMR. Despite the transformative potential, challenges remain, including cost, standardization, and technical limitations. However, with continued technological advancement, decreasing costs, progress in standardization, and the accumulation of robust clinical evidence, the role of NGS in infectious disease management is poised to expand significantly. Future efforts must focus on technical optimization, the establishment of universal standards, the refinement of clinical application strategies, and fostering multidisciplinary collaboration. Through these endeavors, the full clinical translation of NGS technologies will be realized, ushering in an era of precision medicine for infectious diseases that delivers faster, more accurate, and individualized patient care, ultimately improving clinical outcomes and optimizing healthcare resource allocation on a global scale.

## Figures and Tables

**Table 1:** Comparison among mNGS, mp-NGS and hc-tNGS.

Features	mNGS (Metagenomic NGS)	mp-tNGS (Multiplex PCR-Based tNGS)	hc-tNGS (Hybridization Capture tNGS)
**Technology**	Untargeted sequencing, no targeted enrichment	Amplification of target regions using multiplex PCR primers	Capture of target sequences using probe hybridization
**Range**	Theoretically all microorganisms in the sample (bacteria, fungi, viruses, parasites)	Pathogens and resistance genes corresponding to predefined primers (typically tens–hundreds of species)	Pathogens covered by predefined probes (can simultaneously enrich thousands or even tens of thousands of targets)
**Advantages**	No need to pre-select targets; can detect unknown or rare pathogens; broadest coverage	High sensitivity (for targeted pathogens); simple workflow; fastest turnaround; relatively low cost	Broad target coverage (wider than mp-tNGS); suitable for complex infections or large-scale screening; enables comprehensive analysis of resistance genes and virulence factors
**Disadvantages**	High cost; significant host DNA interference; complex data analysis; relatively low sensitivity for low-abundance pathogens	Detection limited to the primer design panel; cannot detect pathogens outside the panel; potential amplification bias due to primer competition	More complex workflow than mp-tNGS
**Application Scenarios**	Difficult or critical infections; emerging infectious disease tracing; research exploration	Rapid screening (e.g., emergency settings); known specific syndromes (e.g., respiratory infections); resistance gene detection with clear targets	Broad-spectrum syndrome screening (e.g., CNS infections, bloodstream infections); scenarios requiring comprehensive pathogen and resistance gene profiling

**Table 2:** Data of mNGS Sensitivity.

Infection Type	Sensitivity	Sensitivity of Other Methods
Central Nervous System Infections	Bacterial 73.3%; Cryptococcal 76.9%; Aspergillus 80%; Tuberculous 66.7–78.3% [38]	-
Respiratory Infections	pulmonary tuberculosis 100% [69]	15.96% by AFB smear method; 40.22% by MTB culture method; 41.67% by TB-DNA
Bloodstream Infections	50.7% [75]	35.2% by culture
Urinary Tract Infections	81.4% [97]	-
	100% [98]	40.0% by culture
Spinal Infections	82.3% [124]	17.5% by culture

**Table 3:** Data of tNGS sensitivity.

Infection Type	Sensitivity	Sensitivity of Other Methods
**Central Nervous System Infections**	70.8% [55]	41.7% by mNGS
**Postoperative CNS Infections (Pediatric)**	81.8% [60]	13.6% by culture
**Respiratory Infections**	pulmonary tuberculosis 77.66% [69]	15.96% by AFB smear method; 40.22% by MTB culture method; 41.67% by TB-DNA

**Table 4:** Comparison between mNGS and tNGS.

Feature	mNGS	tNGS
**TAT**	24 h	24–48 h
**Costs**	High	Relatively low
**Targets covered**	All microorganisms (unbiased)	Limited to predefined panel
**A priori knowledge of pathogens needed**	Not required	required
**Bioinformatic complexity**	High	Moderate

## Data Availability

No new data were created or analyzed in this study. Data sharing is not applicable to this article.
